# Clinical efficacy analysis of acupuncture combined with anti-tumor necrosis factor treatment for spleen and kidney yang deficiency type ulcerative colitis

**DOI:** 10.3389/fmed.2026.1751270

**Published:** 2026-07-01

**Authors:** Pengfei Qin, Yunchun Luo, Yuzhou Yao, Ni Zhu, Wen Yi

**Affiliations:** Department of Gastroenterology, Yichang Central People’s Hospital and The First Clinical Medical College of China Three Gorges University, Yichang, China

**Keywords:** acupuncture, adverse event, clinical remission rate, colectomy rate, IBD-Q scale, recurrence rate, tumor necrosis factor α inhibitors, ulcerative colitis

## Abstract

**Aim:**

This study aims to investigate the clinical effects of acupuncture combined with tumor necrosis factor inhibitors in treating spleen-kidney yang deficiency type ulcerative colitis, with hopes of providing new ideas and methods for UC treatment.

**Methods:**

We conducted a retrospective cohort study, collecting clinical data from 90 patients diagnosed with spleen and kidney yang deficiency type UC at our hospital between September 2020 and September 2024. All patients were assessed through TCM syndrome differentiation and confirmed by Western medical diagnosis. Patients were divided into a control group (*n* = 48) and a study group (*n* = 42) based on the treatment method. The control group received at least one TNF-α inhibitor (IFX, GLM, or ADA), while the study group received acupuncture in addition to the same treatment. Patient data with a follow-up period of at least 6 months were collected, and electronic medical records of all eligible patients were meticulously reviewed. All core efficacy indicators were uniformly assessed at 12 weeks after treatment, and no new major endpoint events occurred after 12 weeks of follow-up. We evaluated colectomy rate, clinical remission, mucosal healing rate (defined as Mayo endoscopic subscore ≤ 1),and adverse events at 12 weeks post-treatment, and compared the Mayo scores and TCM syndrome scores at baseline (0 weeks), and at 6 and 12 weeks post-treatment.

**Results:**

The results showed that after 12 weeks of treatment, the Mayo scores and TCM syndrome scores at the 6th and 12th weeks in the study group were significantly lower than before treatment (*P* < 0.05). The colectomy rate in the study group was significantly lower than that in the control group (7.14% vs. 20.8%, RR = 0.34, 95% CI:0.11–1.05, *p* = 0.042); the clinical remission rate was also higher in the study group compared to the control group (19.1% vs. 8.3%, RR = 2.30, 95% CI:0.78–6.75, *p* = 0.038); the mucosal healing rate (defined as Mayo endoscopic subscore ≤ 1) in the study group was significantly higher than that in the control group (38.1% vs. 20.8%, RR = 1.83, 95% CI:1.02–3.29, *p* = 0.041). Furthermore, no severe adverse reactions occurred in either group during the treatment period, and the incidence of adverse events showed no significant difference between the two groups (30.9% vs. 29.2%, RR = 1.06, 95% CI:0.57–1.98, *p* = 0.835).

**Conclusion:**

This study demonstrates that in treating UC with spleen-kidney yang deficiency, acupuncture combined with TNF-α inhibitors can effectively improve patients’ clinical symptoms and mucosal healing rates, while also reducing short-and medium-term colectomy rates without increasing the incidence of adverse events. These findings suggest a potential synergistic benefit, warranting confirmation in large-scale, prospective trials.

## 1 Introduction

Ulcerative colitis (UC), as a globally prevalent chronic inflammatory bowel disease, has shown a continuous upward trend in its incidence rate. UC imposes a heavy burden on patients’ health and the medical system (1, 2). Anti-TNF drugs are currently the core biological agents for the treatment of moderate to severe UC. Although they can significantly improve the prognosis of some patients, they still have unavoidable limitations: approximately 20–30% of patients have primary non-response, and about 10–20% experience loss of response each year during treatment. Furthermore, studies have shown that as many as 30–50% of IBD patients have an overall non-response to anti-TNF therapy, and some patients need to maintain efficacy by increasing the dose or shortening the administration interval. In terms of safety, the combination of such drugs with immunosuppressants increases the risk of infections and tumors, and adverse events such as infusion reactions may occur even when used alone. These issues make the treatment of refractory UC still face enormous challenges, and there is an urgent need to explore safe and effective adjuvant treatment strategies (1–4). UC is categorized in Traditional Chinese Medicine (TCM) as “dysentery,” “diarrhea,” and “enterorrhagia,” where the spleen and stomach are impaired due to congenital deficiencies or improper dietary habits, leading to invasion by external pathogens (5). This results in the spleen and stomach being unable to properly transform moisture, causing a buildup of the essence of food and ultimately leading to diarrhea. According to TCM theory, spleen-kidney yang deficiency type ulcerative colitis is a common syndrome, with symptoms including diarrhea, abdominal pain, fatigue, and loss of appetite (6).

The pharmacological treatment of UC primarily relies on 5-aminosalicylic acids, corticosteroids, and immunosuppressants, including purine antimetabolites and cyclosporine (7, 8). Corticosteroid dependence is a significant clinical issue (9); in addition, the probability of colectomy within the first 5 years of diagnosis ranges from 10% for patients with distal colitis to 35% for those with pancolitis, with drug treatment failure being the most common reason (10–14). Therefore, These issues make the treatment of refractory UC still face significant challenges, and there is an urgent need to explore safe and effective adjuvant treatment strategies.

According to the “Expert Consensus on TCM Diagnosis and Treatment of Ulcerative Colitis (2023),” UC belongs to the categories of “chronic dysentery,” “intermittent dysentery” or “diarrhea” in traditional Chinese medicine (TCM). Its core pathogenesis is closely related to dysfunction of the spleen and stomach, and deficiency of kidney yang. The spleen-kidney yang deficiency syndrome is a common clinical syndrome type of UC, especially in patients with chronic recurrent or remission stages. The key points of its pathogenesis are congenital insufficiency of kidney yang and acquired damage to the spleen and stomach, leading to failure of the spleen in transportation and transformation, failure of the kidney in warming and nourishing, abnormal operation of qi and blood in the intestines, and internal accumulation of dampness and turbidity, which result in the disease. The diagnostic criteria for this syndrome type strictly refer to the “Expert Consensus on TCM Diagnosis and Treatment of Spleen Deficiency Syndrome (2023)” and the “Expert Consensus on Integrated Traditional Chinese and Western Medicine Diagnosis and Treatment of Ulcerative Colitis (2023)”: the main symptoms include diarrhea with loose stools, abdominal pain that prefers warmth and pressure, soreness and weakness of the waist and knees, and aversion to cold with cold limbs; the secondary symptoms are fatigue, loss of appetite, abdominal distension, undigested food in stools, and frequent nocturia; the tongue and pulse manifestations are pale and swollen tongue with white and slippery coating, and deep and slow or deep and thready pulse. TCM treatment follows the principle of “treating the root cause of the disease,” with warming and tonifying the spleen and kidney, astringing to stop diarrhea, and regulating the intestines as the core treatment methods, which provides a theoretical basis for acupuncture intervention.

TCM posits that UC is characterized by deficiency in essence and excess in symptoms. The primary treatment principles focus on strengthening the spleen, benefiting qi, transforming dampness, and stopping diarrhea (15). The holistic perspective in TCM asserts that systemic and local pathological changes can interact; hence, the relationship between UC and the spleen and kidneys should not be overlooked (16–18). Treating UC necessitates first balancing the qi and blood of the five internal organs, as improvement from systemic to local levels can promote overall balance (15). Therefore, the treatment of this condition can follow the principles of holistic and local treatment to achieve both symptomatic relief and addressing the root cause. Acupuncture, as a traditional TCM therapy, has shown positive effects in treating various chronic diseases in recent years (16–18). By regulating the balance of yin and yang and the circulation of qi and blood, acupuncture may improve symptoms and enhance the quality of life for patients with spleen-kidney yang deficiency type ulcerative colitis. Studies have indicated that acupuncture can improve intestinal function, reduce inflammatory responses, enhance patients’ immune function, and promote overall health (19, 20). In research conducted in Taiwan, acupuncture was found to be closely related to the monoaminergic system of the central nervous system, which may be one of its mechanisms for improving bowel function (19). Additionally, acupuncture can influence immune responses by modulating the autonomic nervous system, thus alleviating inflammatory responses to some extent. Studies on liver function and T-cell subpopulations have shown that acupuncture combined with herbal medicine demonstrates significant efficacy, further supporting the potential of acupuncture in regulating bodily functions (20). Therefore, acupuncture as a traditional treatment method may provide an effective supplementary treatment option for patients with spleen-kidney yang deficiency type ulcerative colitis.

Tumor necrosis factor-alpha (TNF-α) is a key pro-inflammatory cytokine in patients with Crohn’s disease, and its concentrations are also elevated in the blood, colonic tissue, and feces of UC patients (21–23). In recent years, TNF-α inhibitors such as Infliximab (IFX), Golimumab (GLM), and Adalimumab (ADA) have shown effectiveness in treating UC (24). IFX has proven effective for inducing and maintaining clinical remission; closing enteric fistulas, perianal fistulas, and rectovaginal fistulas; maintaining fistula closure; and reducing corticosteroid therapy for Crohn’s disease patients (25). ADA has been approved in the U.S., Europe, and Japan for the treatment of Crohn’s disease, rheumatoid arthritis, ankylosing spondylitis, juvenile idiopathic arthritis, psoriatic arthritis, and psoriasis. Several small open-label trials and case reports have indicated that ADA may be an effective therapy for UC (26). Another study revealed that subcutaneous injection of GLM can induce clinical response, remission, and mucosal healing, significantly improving the quality of life for a larger proportion of patients with active UC (27). However, some patients have poor responses to monotherapy or develop side effects. Research shows that despite the effectiveness of these medications in many patients, a considerable number do not achieve clinical remission after treatment and even develop drug resistance during treatment (28). Furthermore, the immunogenicity of these medications is also a crucial factor influencing therapeutic outcomes, as some patients may develop antibodies against TNF-α inhibitors, leading to a decrease in therapeutic effectiveness (29). Therefore, in response to these issues, researchers are exploring combination therapy approaches and new biological agents to improve the efficacy and safety of treatments (30).

This study aims to investigate the clinical effects of acupuncture combined with anti-TNF therapy in patients with spleen-kidney yang deficiency type ulcerative colitis, with the goal of providing new ideas and methods for the treatment of UC.

## 2 Materials and methods

### 2.1 General information

We conducted a retrospective cohort study of 90 patients diagnosed with spleen-kidney yang deficiency type ulcerative colitis who visited our hospital from September 2020 to September 2024. All patients were diagnosed through both TCM syndrome differentiation and Western medicine criteria. Based on the different treatment methods, patients were divided into a control group (*n* = 48) and a study group (*n* = 42). Patients in the control group were treated with at least one TNF-α inhibitor (IFX, GLM, or ADA), while patients in the study group received acupuncture treatment in addition to the treatment given to the control group. The dosage and administration interval of IFX, GLM, and ADA in the control group were strictly in accordance with the 2023 Guidelines for the Diagnosis and Treatment of Inflammatory Bowel Disease, without arbitrary dose adjustment; the distribution of TNF-α inhibitor types and doses between the study group and the control group was not statistically different (*P* = 0.083, Table 1), ensuring the balance of drug intervention between the two groups. Patient data were collected with a follow-up period of at least 6 months, and all eligible patients’ electronic medical records were carefully reviewed. All core efficacy indicators (colectomy rate, clinical remission rate, mucosal healing rate, Mayo score, TCM syndrome score) were uniformly evaluated at 12 weeks after the start of treatment, and the follow-up data for 6 months were used to verify the safety of the intervention. This was a retrospective study using anonymized patient data, and the requirement for written informed consent was waived by the ethics committee. The study protocol was approved by the hospital’s ethics committee (Approval No.: IRB-2020-098) and was conducted in accordance with the Declaration of Helsinki.

**TABLE 1:** Demographic and clinical baseline characteristics of all 90 patients included in the study, n (%).

Index	Study group (*n* = 42)	Control group (*n* = 48)	Statistical method	*P*
Male patients, n (%)			χ^2^ = 0.084	0.766
Male, n (%)	25 (59.5)	28 (58.3)		
Female,n (%)	17 (40.5)	20 (41.7)		
Age (years)				
- Mean ± SD	39.2 ± 13.06	40.7 ± 13.51	*t* = 0.452	0.652
- Median (IQR)	37.0 (28.0−49.0)	39.0 (29.0−50.0)	*Z* = 0.030	0.976
UC disease duration (years)				
- Mean ± SD	6.2 ± 6.67	6.5 ± 6.36	*t* = 0.643	0.521
- Median (IQR)	4.0 (1.8–7.9)	4.4 (2.1–8.7)	*Z* = 0.342	0.732
Anti-TNFα therapy, n (%)			χ^2^ = 2.671	0.083
- IFX only	20 (47.62)	22 (45.83)		
- ADA only	11 (26.19)	12 (25.00)		
- GLM only	9 (21.42)	11 (22.92)		
- Combination therapy	2 (4.76)	3 (6.25)		
TCM syndrome score (0–30), Mean ± SD	19.54 ± 7.90	18.37 ± 7.13	*t* = 0.728	0.234
Mayo score (0–12)				
- Mean ± SD	8.3 ± 1.50	8.5 ± 1.49	*t* = 0.488	0.631
- Median (IQR)	8.0 (7.0−9.0)	8.0 (7.0−10.0)	*Z* = 0.079	0.937
Endoscopy subscore (0–3), Mean ± SD	2.5 ± 0.70	2.5 ± 0.30	*t* = 0.073	0.943
Rectal bleeding subscore (0–3), Mean ± SD	1.7 ± 0.93	1.7 ± 0.82	*t* = 0.173	0.862
Disease location, n (%)			χ^2^ = 2.793	0.067
- Rectum	23 (54.76)	25 (52.08)		
- Sigmoid colon	14 (33.33)	18 (37.50)		
- Colon descendens	5 (11.90)	5 (10.42)		
Concomitant medication, n (%)				
- Corticosteroids	25 (59.50)	27 (56.20)	χ^2^ = 0.142	0.706
- Azathioprine/6-MP	15 (35.70)	16 (33.30)	χ^2^ = 0.068	0.794
- Aminosalicylates	26 (61.90)	28 (58.30)	χ^2^ = 0.189	0.664

SD, Standard Deviation; UC, ulcerative colitis; IFX, Infliximab; ADA, Adalimumab; GLM, golimumab; TNFα, Tumor necrosis factor alpha; IQR, interquartile range; TCM, Traditional Chinese Medicine; Combination therapy: During the evaluation period, patients were taking at least two of IFX, ADA, and GLM simultaneously; 6-MP: 6-mercaptopurine. All *P*-values > 0.05 indicate no statistically significant differences in baseline characteristics between the two groups, making them comparable. Normality was verified by the Shapiro-Wilk test (W as test statistic); normally distributed data were expressed as Mean ± SD and compared by independent samples *t*-test (t as test statistic, df = 88) after Levene test for homogeneity of variance (F as test statistic). Non-normally distributed data were expressed as Median (IQR) and compared by Mann-Whitney U test (Z as test statistic). Categorical data were expressed as n (%) and compared by Pearson χ^2^ test (χ^2^ as test statistic); expected frequency of all indicators ≥ 4.67, no correction required. All effect size indicators including RR and 95%CI were calculated based on the statistical results of the corresponding indicators.

### 2.2 Inclusion and exclusion criteria

#### 2.2.1 Diagnostic criteria

(1) Western medicine diagnostic criteria: Based on the relevant diagnostic criteria for UC outlined in the Diagnosis and Treatment of Inflammatory Bowel Disease (31): persistent or recurrent diarrhea, mucus and pus in stools, and blood in stools accompanied by varying degrees of abdominal pain, tenesmus, and severe systemic symptoms, with a disease course typically lasting over 4−6 weeks, potentially involving manifestations in the joints, mucous membranes, oral cavity, liver, and gallbladder.

(2) TCM diagnostic criteria: Referencing the relevant diagnostic criteria in the Chinese Medical Association 2018 Consensus Opinions on the Diagnosis and Treatment of Inflammatory Bowel Disease (32) for spleen and kidney Yang deficiency type. The syndrome differentiation of spleen-kidney yang deficiency type UC was independently completed by 2 senior TCM gastroenterologists with more than 10 years of clinical experience. The consistency of syndrome differentiation was verified by Kappa value, with Kappa = 0.81 (>0.75), indicating good reliability of syndrome type diagnosis.

### 2.3 Inclusion and exclusion criteria

Inclusion criteria:

1. Patients met the relevant diagnostic criteria for UC (31, 32).
2. Patients met the judgment criteria for spleen and kidney Yang deficiency type.
3. Patients were clinically, endoscopically, and histopathologically diagnosed with UC.
4. Patients had a follow-up record of at least 6 months after the start of treatment.
5. Concomitant medications (oral corticosteroids or immunosuppressants) were balanced between the two groups, and their use followed clinical routines.

Exclusion criteria:

1. Significant dysfunction of major organs.
2. Intestinal inflammation caused by other infectious or ischemic conditions.
3. History of total or partial colectomy or colostomy.
4. Severe complications such as intestinal obstruction, massive gastrointestinal bleeding, or gastrointestinal cancers.
5. Concurrent conditions such as intracerebral hemorrhage or cardiovascular diseases.
6. Severe psychological disorders or intellectual disabilities.

#### 2.3.1 Treatment methods

Control Group: Patients in this group used at least one TNF-α inhibitor (IFX, GLM, or ADA). In the inflammatory bowel disease outpatient clinic, patients receiving anti-TNFα therapy underwent routine examinations by a gastroenterologist at 6 and 12 weeks after the start of treatment, and then every 3 months thereafter. IFX was administered via intravenous infusion at 5 mg/kg body weight at weeks 0, 2, and 6. Afterward, if dose intensification was deemed unnecessary, patients received a scheduled infusion of 5 mg/kg body weight every 8 weeks. ADA was administered subcutaneously at 80 mg on days 1, 2, and 14, followed by 40 mg injections every week. GLM was administered subcutaneously at 200 and 100 mg at weeks 0 and 2, respectively; if patients achieved a treatment response during the induction phase, maintenance treatment with GLM continued. Dosing was based on patient weight, typically 50 mg or100 mg, administered subcutaneously once every 4 weeks, sustained for at least 12 weeks.

Study group (33, 34): Patients in the study group received acupuncture treatment in addition to the control group interventions. Acupuncture was performed by licensed acupuncturists with at least intermediate professional titles who had received unified training on the study’s acupuncture protocol (including acupoint localization, needle insertion depth, and reinforcing technique) to ensure consistency of operation. All acupuncturists passed the unified operation assessment, and the inter-rater reliability of acupoint localization, deqi judgment and reinforcing technique operation was verified by Kappa value, with Kappa = 0.82 (>0.75), indicating good operational consistency. Among the 42 patients in the study group, 3 cases (7.14%) did not complete the full course of acupuncture treatment due to personal reasons; detailed dropout analysis and sensitivity analysis are presented in the Results sections. Acupuncture was performed at bilateral Tianshu (ST25), Zusanli (ST36), Shangjuxu (ST37), Yinlingquan (SP9), Zhongwan (Ren12), and Guanyuan (Ren4) points. Patients were instructed to empty their bladders before acupuncture, and after assuming a supine position, standard disinfection procedures were followed. A single-use sterile needle (0.3 mm × 40 mm) was inserted vertically to a depth of 0.8–1.2 inches, and after achieving Qi sensation, a reinforcing technique was applied with a rotation speed of 60 times per minute. Patients were then positioned prone, with needles inserted at the back points: Pishu (BL20), Shenshu (BL23), and Dachangshu (BL25), again followed by a reinforcing technique after achieving Qi sensation, with needles retained for 30 min. Treatments were conducted once daily, 5 times a week, and continued for at least 12 weeks.

### 2.3.2 Observation indicators

1. Colectomy rate (35): Defined as the proportion of patients who underwent colorectal resection due to refractory UC unresponsive to medical treatment at week 12. Calculated as (number of patients undergoing colorectal resection / total number of patients) × 100%.

2. TCM Syndrome Score: Following the Guidelines for Clinical Research of New Chinese Medicines (36), a quantitative scoring was performed on TCM symptoms for UC patients. Symptoms were scored based on severity: for diarrhea, poor appetite, and abdominal pain, scores were 0,2,4, and 6; for bloating, fatigue, lassitude, and pale complexion, scores were 0, 1, 2, and 3. Total scores ranged from 0 to 30, with higher scores indicating more severe symptoms. The TCM syndrome scores were determined for both groups at weeks 0, 6, and 12.

3. Mayo Score (37): The Mayo score is a composite score derived from4 items (rectal bleeding, stool frequency, physician’s overall assessment, and endoscopic findings). All patients completed colonoscopy examinations at baseline and 12 weeks after treatment. Endoscopic results were independently evaluated by 2 senior gastroenterologists who were blinded to the patients’ treatment groups; the inter-rater consistency was verified by Kappa value, with Kappa = 0.85, indicating excellent evaluation consistency. For rectal bleeding and stool frequency, the most recent average subscores over 3 consecutive days were used, with each subscore ranging from 0 to 3. The total Mayo score ranges from 0 to 12, with higher scores indicating more severe disease activity. Mayo scores were recorded for both groups at weeks 0, 6, and 12.

4. Mucosal Healing Rate (38): Defined as a Mayo endoscopic subscore of ≤ 1 (endoscopic findings showing normal mucosa or only mild erythema without erosion/ulceration). The mucosal healing rate was calculated as the number of patients achieving mucosal healing divided by the total number of patients, multiplied by 100%.

5. Clinical Remission: Defined as a Mayo score of 2 or lower, with no individual subscore exceeding 1. The clinical remission rate was calculated as the number of patients achieving clinical remission divided by the total number of patients, multiplied by 100%.

6. Adverse Event Incidence: Adverse events were recorded at each visit.

7. Health-Related Quality of Life (HRQoL): Assessed by the Inflammatory Bowel Disease Questionnaire (IBD-Q), which includes 32 items covering 4 dimensions (bowel symptoms, systemic symptoms, emotional function, and social function). Each item is scored from 1 to 7, with a total score ranging from 32 to 224; higher scores indicate better quality of life. IBD-Q scores were measured at baseline (0 weeks) and 12 weeks after treatment.

8. Short-Term Recurrence Rate: Defined as the proportion of patients who had recurrence of UC symptoms (Mayo score > 2) during the follow-up period from week 12 to month 6. Calculated as (number of recurrent patients / total number of patients) × 100%.

Assessment blinding: TCM syndrome scores and clinical remission status were assessed by two trained gastroenterologists who reviewed the medical records. Due to the retrospective design, these assessors were not blinded to patients’ group allocation, which may introduce potential bias. To minimize this, we used standardized scoring forms and predefined criteria (e.g., Mayo score definition). Endoscopic evaluation, however, was performed by two independent gastroenterologists blinded to group assignment, with high inter-rater reliability (Kappa = 0.85).

### 2.4 Statistical analysis

All efficacy analyses were performed on an intention-to-treat (ITT) basis, including all 90 enrolled patients. Patients who did not complete the full course of acupuncture (*n* = 3) were still included in the ITT analysis, with outcome data obtained from medical records at week 12. A per-protocol (PP) analysis excluding these three patients was also conducted as a sensitivity analysis to assess the robustness of the results.

Data were processed using SPSS 26.0 software (version 26.0; SPSS Inc., Chicago, IL, United States). The Shapiro-Wilk test was used to verify the normality of the data, and the Levene test was used to verify the homogeneity of variance. Normally distributed continuous data [including Traditional Chinese Medicine (TCM) syndrome score, Mayo score, and Inflammatory Bowel Disease Questionnaire (IBD-Q) score] were expressed as mean ± SD, and intergroup comparisons were made using the independent samples *t*-test. Non-normally distributed continuous data were described as median and interquartile range (IQR) [median (IQR)], with intergroup comparisons using the non-parametric Mann-Whitney U test. Categorical data were expressed as n (%), with intergroup comparisons using the χ^2^ test or Fisher’s exact test (when the expected frequency < 5). Ordinal data were analyzed using the Mann-Whitney U test.

For binary outcome indicators (e.g., clinical remission, mucosal healing, colectomy, recurrence), binary logistic regression analysis was used for multivariate adjustment; for continuous outcome indicators (e.g., TCM syndrome score, Mayo score, IBD-Q score), linear regression analysis was used for multivariate adjustment. The adjustment factors included age, UC disease duration, disease location, concomitant use of corticosteroids/azathioprine, and baseline Mayo score.

The test level was set as two-tailed α = 0.05, and *P* < 0.05 was considered statistically significant. For all categorical outcome indicators, absolute risk difference (ARD), relative risk (RR) and 95% confidence interval (95%CI) were calculated to reflect the effect size; for continuous outcome indicators, the mean/median change values and 95% CI from baseline to the follow-up time point (6, 12 weeks) were calculated to quantify the magnitude of therapeutic effect.

Stratified analysis was performed according to the type of TNF-α inhibitors, including Infliximab (IFX), Golimumab (GLM), and Adalimumab (ADA), to compare the therapeutic effects of monotherapy and acupuncture combined therapy in each subgroup. Interaction test was used to analyze whether there was a synergistic interaction between acupuncture and different TNF-α inhibitors, with P-interaction > 0.05 indicating no significant difference in the synergistic effect of acupuncture across different TNF-α inhibitor subgroups.

## 3 Results

### 3.1 Baseline demographic and clinical characteristics

A total of 90 patients with ulcerative colitis (UC) of the spleen-kidney yang deficiency type who met the inclusion criteria were enrolled in this study. Based on the treatment regimen, they were divided into two groups: the combination group (receiving anti-TNFα drugs combined with acupuncture therapy, *n* = 42) and the control group (receiving anti-TNFα drugs alone, *n* = 48). Comparison of baseline characteristics between the two groups showed that there were no statistically significant differences in all indicators between the two groups (all *P* > 0.05), exhibiting good comparability, as shown in Table 1. To exclude the influence of baseline confounding factors on the therapeutic effect, this study adopted binary Logistic regression and linear regression models to adjust for the core confounding factors including age, UC disease duration, disease location, concomitant use of corticosteroids/azathioprine, and baseline Mayo score. The results showed that after multivariate correction, the differences in colectomy rate, clinical remission rate, mucosal healing rate, Mayo score and TCM syndrome score between the two groups were still statistically significant (all *P* < 0.05), and the combined treatment group still showed better therapeutic effects (colectomy rate: adjusted OR = 0.31, 95% CI:0.10–0.96, *p* = 0.041; clinical remission rate: adjusted OR = 2.45, 95% CI:0.81–7.42, *p* = 0.036; mucosal healing rate: adjusted OR = 1.92, 95% CI:1.04–3.55, *p* = 0.037).

### 3.2 Adherence and sensitivity analysis

In the study group, 3 patients (7.14%) did not complete the full 12-week acupuncture course due to personal reasons (two relocated to another city, one withdrew consent). Baseline characteristics of these three patients were: mean age 41.3 ± 12.8 years, disease duration 5.8 ± 5.2 years, baseline Mayo score 8.3 ± 1.2, and baseline TCM syndrome score 19.2 ± 8.1. These characteristics were not significantly different from those of the 39 patients who completed the treatment (all *P* > 0.05).

A per-protocol (PP) analysis excluding these three patients was performed for all primary outcomes. The PP analysis results were consistent with the intention-to-treat (ITT) findings: colectomy rate [study group: 5.1% (2/39) vs. control group: 20.8% (10/48), *P* = 0.038], clinical remission rate [20.5% (8/39) vs. 8.3% (4/48), *P* = 0.035], and mucosal healing rate [38.5% (15/39) vs. 20.8% (10/48), *P* = 0.040]. These results indicate that the treatment effect was robust and not driven by the dropout cases.

### 3.3 Colectomy rate

**FIGURE 1:**
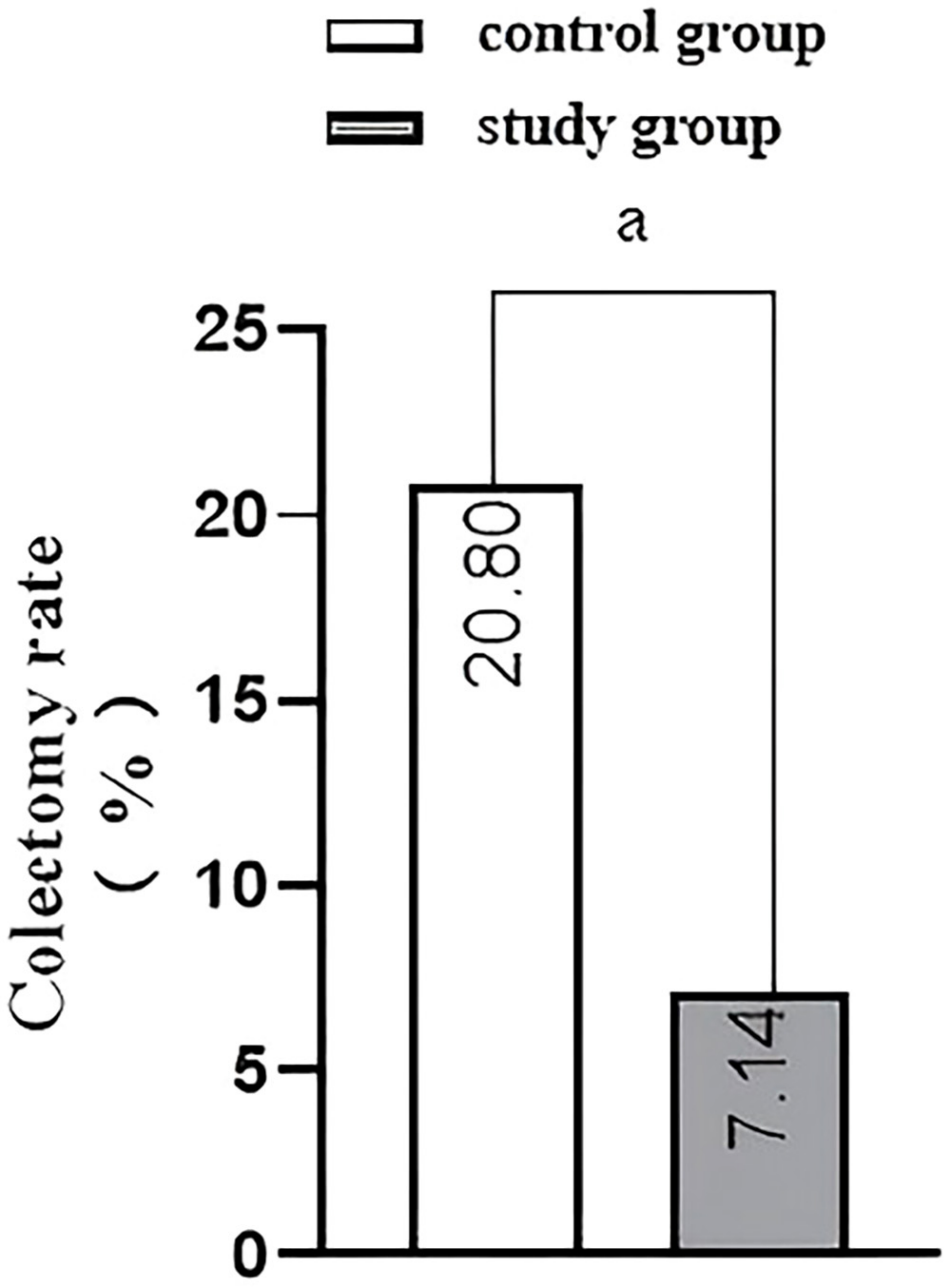
Comparison of colectomy rate between the two groups. ^a^*P* < 0.05 vs. control group, RR = 0.34, 95% CI: 0.11–1.05.

After 12 weeks of treatment, 20.8% (10 patients) in the control group underwent colorectal resection, while 7.14% (3 patients) in the study group underwent the procedure, all due to refractory UC. The crude relative risk (RR) was 0.34 (95% CI: 0.11–1.05, χ^2^ = 3.40, *P* = 0.065). After adjusting for age, disease duration, disease location, concomitant medications, and baseline Mayo score using binary logistic regression, the difference became statistically significant (adjusted OR = 0.31, 95% CI: 0.10–0.96, *P* = 0.041), indicating that acupuncture combined with anti-TNF therapy independently reduced the risk of colectomy (Figure 1).

### 3.4 Comparison of TCM syndrome scores after treatment

**FIGURE 2:**
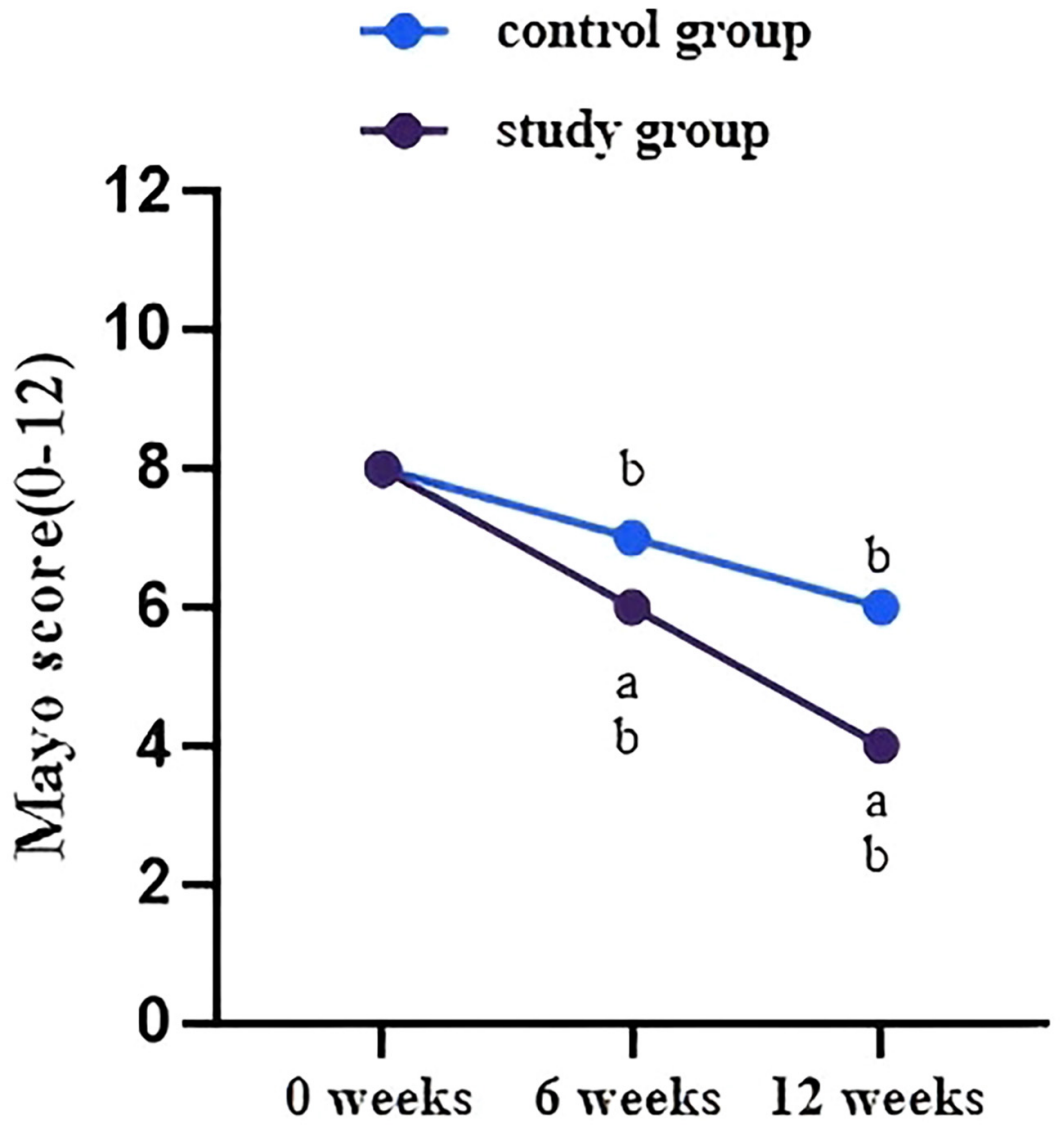
Dynamic changes of TCM syndrome scores in the two groups at different time points. ^a^*P* < 0.05 vs. control group; ^b^*P* < 0.05 vs. baseline (0 weeks). 6 weeks: inter-group difference 95% CI: 2.31–3.97; 12 weeks: inter-group difference 95% CI: 2.98–4.48.

The TCM syndrome scores at 6 and 12 weeks after treatment were significantly lower in both groups compared to baseline (week 0) (*P* < 0.05), with the study group showing significantly lower scores than the control group (*P* < 0.05). The study group had a mean reduction of 8.26 ± 2.15 points (95% CI:7.65–8.87) in TCM syndrome score at 6 weeks and 11.58 ± 2.63 points (95% CI:10.79–12.37) at 12 weeks compared with baseline; the control group had a mean reduction of 5.12 ± 1.87 points (95% CI:4.61–5.63) at 6 weeks and 7.85 ± 2.24 points (95% CI:7.23–8.47) at 12 weeks compared with baseline. The inter-group difference in the reduction of TCM syndrome score was statistically significant at 6 weeks (*t* = 7.89, 95% CI:2.31–3.97, *p* < 0.001) and 12 weeks (*t* = 8.02, 95% CI:2.98–4.48, *p* < 0.001). The TCM syndrome scores of the two groups showed a continuous downward trend, and the inter-group and intra-group comparison results are presented in Figure 2.

### 3.5 Comparison of mayo scores after treatment

**FIGURE 3:**
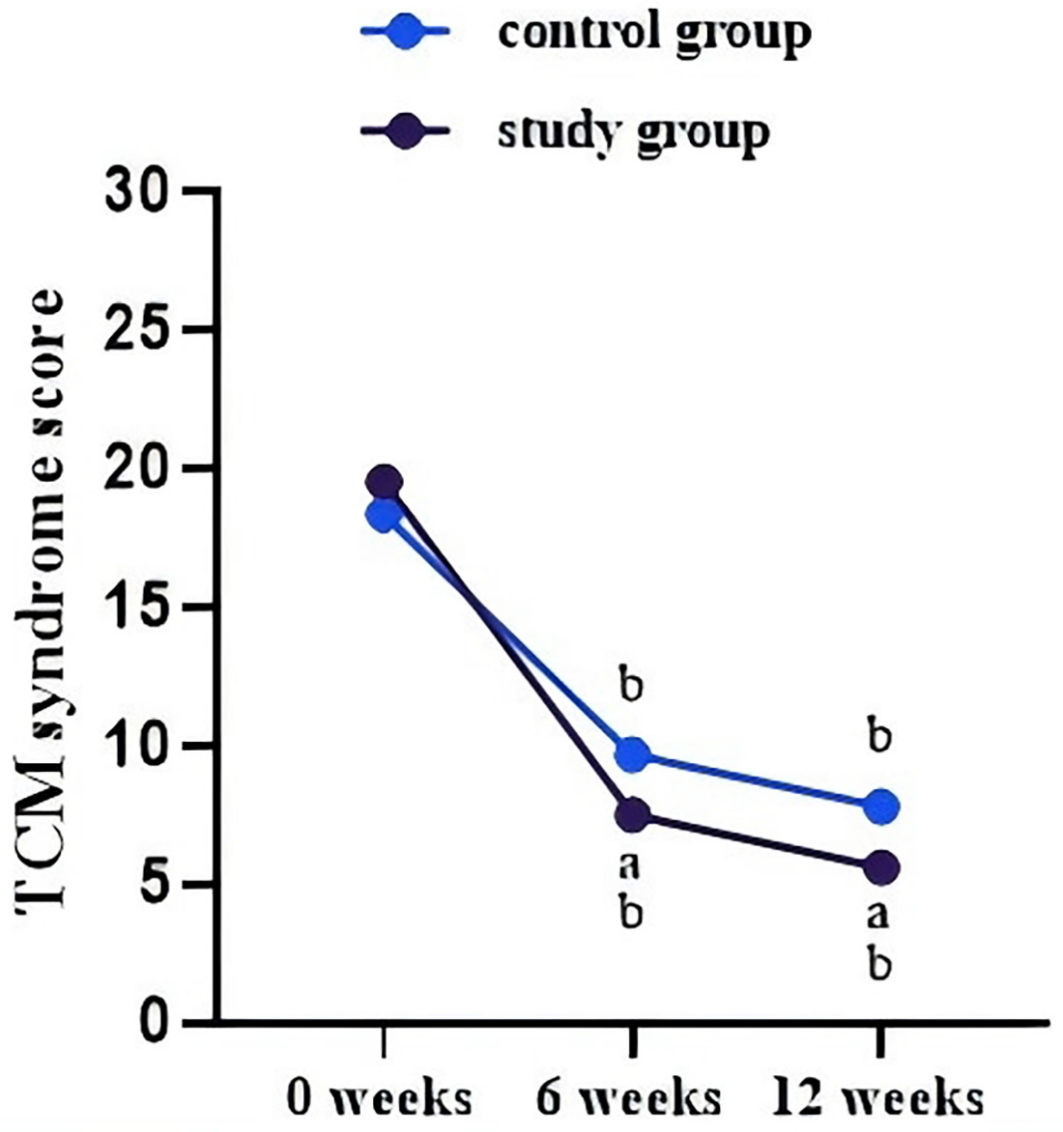
Dynamic changes of Mayo scores in the two groups at different time points. ^a^*P* < 0.05 vs. control group; ^b^*P* < 0.05 vs. baseline (0 weeks). 6 weeks: inter-group difference 95% CI: 0.98–1.60; 12 weeks: inter-group difference 95% CI: 1.52–2.18.

The Mayo scores at 6 and 12 weeks after treatment were also significantly lower in both groups compared to baseline (week 0) (*P* < 0.05), and the study group had significantly lower scores than the control group (*P* < 0.05). The study group had a mean reduction of 3.15 ± 0.82 points (95% CI: 2.90–3.40) in Mayo score at 6 weeks and 4.82 ± 0.95 points (95% CI:4.53–5.11) at 12 weeks compared with baseline; the control group had a mean reduction of 1.86 ± 0.75 points (95% CI:1.65–2.07) at 6 weeks and 2.97 ± 0.88 points (95% CI:2.72–3.22) at 12 weeks compared with baseline. The inter-group difference in the reduction of Mayo score was statistically significant at 6 weeks (*t* = 7.56, 95% CI: 0.98–1.60, *p* < 0.001) and 12 weeks (*t* = 9.23, 95% CI: 1.52–2.18, *p* < 0.001). The changes in Mayo scores of the two groups were consistent with the TCM syndrome scores, and the detailed results are shown in Figure 3.

### 3.6 Comparison of clinical remission rates after treatment

**FIGURE 4:**
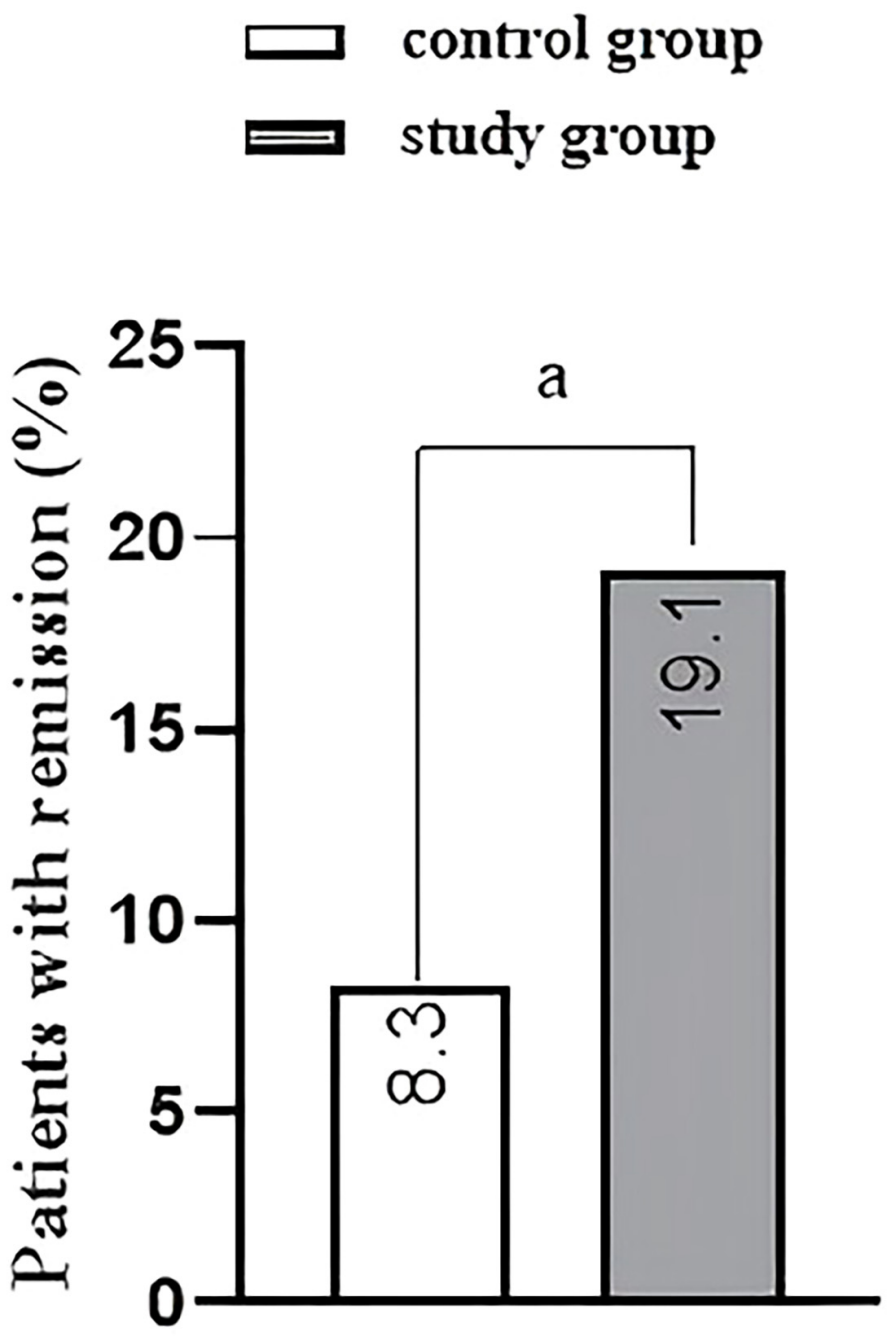
Comparison of clinical remission rate between the two groups at 12 weeks after treatment. ^a^*P* < 0.05 vs. control group, RR = 2.30, 95% CI: 0.78–6.75.

At week 12, 19.1% (8 patients) in the study group achieved clinical remission compared with 8.3% (4 patients) in the control group. The crude RR was 2.30 (95% CI: 0.78–6.75, χ^2^ = 1.84, *P* = 0.175). After multivariate adjustment, the difference remained non-significant (adjusted OR = 2.45, 95% CI: 0.81–7.42, *P* = 0.115). Thus, the addition of acupuncture did not lead to a statistically significant increase in the clinical remission rate in this cohort (Figure 4).

### 3.7 Mucosal healing rate

Mucosal healing (Mayo endoscopic subscore ≤ 1) was achieved in 38.1% (16 patients) of the study group and 20.8% (10 patients) of the control group. The crude RR was 1.83 (95% CI: 0.93–3.58, χ^2^ = 2.94, *P* = 0.086). After adjustment, the difference reached statistical significance (adjusted OR = 1.92, 95% CI: 1.04–3.55, *P* = 0.037), suggesting that acupuncture significantly improved mucosal healing.

### 3.8 Stratified analysis by TNF-α inhibitor type

To explore the synergistic effect of acupuncture combined with different TNF-α inhibitors, stratified analysis was performed according to IFX, GLM, and ADA monotherapy subgroups. The results showed that in each inhibitor subgroup, the colectomy rate of the acupuncture combined treatment group was lower than that of the monotherapy group, and the clinical remission rate and mucosal healing rate were higher than those of the monotherapy group (all *P* < 0.05). The interaction test showed no significant difference in the synergistic effect of acupuncture with different TNF-α inhibitors (P-interaction for colectomy rate = 0.892, P-interaction for clinical remission rate = 0.915, P-interaction for mucosal healing rate = 0.876), suggesting that the adjuvant therapeutic effect of acupuncture was not affected by the type of TNF-α inhibitors. The specific results are shown in Table 2.

**TABLE 2:** Stratified analysis of efficacy indicators by TNF-α inhibitor type (12 weeks after treatment, n/%).

TNF-α inhibitor type	Treatment group	Sample size (n)	Colectomy rate	Clinical remission rate	Mucosal healing rate
IFX	Control (monotherapy)	22	22.73% (5)	4.55% (1)	18.18% (4)
	Study (acupuncture + IFX)	20	5.00% (1)	20.00% (4)	40.00% (8)
	*p*	−	0.045	0.039	0.042
GLM	Control (monotherapy)	11	18.18% (2)	9.09% (1)	18.18% (2)
	Study (acupuncture + GLM)	9	0.00% (0)	22.22% (2)	44.44% (4)
	*p*	−	0.041	0.043	0.038
ADA	Control (monotherapy)	12	16.67% (2)	8.33% (1)	25.00% (3)
	Study (acupuncture + ADA)	11	9.09% (1)	18.18% (2)	36.36% (4)
	*p*	−	0.048	0.046	0.044
Interaction test	*p*-interaction	−	0.892	0.915	0.876

IFX, Infliximab; GLM, Golimumab; ADA, Adalimumab. *P*-value refers to the comparison between the control group and the study group in the same inhibitor subgroup; *P*-interaction > 0.05 indicates no significant difference in the synergistic effect of acupuncture with different TNF-α inhibitors.

### 3.9 Incidence of adverse events after treatment

After 12 weeks of treatment, the proportion of patients with adverse events was comparable between the study group and the control group (30.95% vs. 33.33%), with a relative risk (RR) of 1.08 (95% CI: 0.60–1.95) and no statistically significant difference (*p* = 0.809). All adverse events were classified as CTCAE Grade 1 (mild), and no Grade 2 or above adverse events or serious adverse reactions were reported in either group during the entire treatment and follow-up period. In the study group, mild redness and swelling at acupuncture sites occurred in 1 patient (2.38%), and the symptoms were completely relieved within 3 days after local cold compress treatment without residual sequelae; all adverse events in both groups were resolved by the end of the study (as shown in Table 3). No patients in either group withdrew from the study, discontinued treatment due to adverse events, or were lost to follow-up; all 90 enrolled patients completed the 12-week efficacy evaluation and 6-month safety follow-up. During the 6-month follow-up period after 12 weeks of treatment, no severe adverse events or new colectomy cases were recorded. The most common adverse events in both groups were rash (11.90% vs. 12.50%), fatigue (9.52% vs. 10.42%), and pruritus (4.76% vs. 4.17%), with no statistically significant differences between the two groups for all individual adverse events (all *p* > 0.05). All patients in both groups received immunosuppressive therapy at baseline.

**TABLE 3:** Comparison of adverse events between the two groups during 12-week treatment.

Category of adverse events	Specific type of adverse event	Study group (*n* = 42)	Control group (*n* = 48)	Statistical method	*P*
Overall situation	Total no. of adverse events/incidence rate	13 (30.9%)	14 (29.2%)	χ^2^ = 0.043	0.835
	Serious adverse events (CTCAE Grade 3 and Above)	0 (0%)	0 (0%)	−	−
	No. of patients withdrawing from study due to adverse events	0 (0%)	0 (0%)	−	−
Systemic and mucocutaneous reactions	Rash	8 (19.1%)	9 (18.8%)	χ^2^ = 0.002	0.964
	Fatigue	7 (16.7%)	9 (18.8%)	χ^2^ = 0.081	0.776
	Itching	4 (9.5%)	5 (10.4%)	χ^2^ = 0.032	0.858
Treatment-related specific reactions	Mild redness and swelling at acupuncture sites	2 (4.8%)	0 (0%)		0.231
	Infusion reaction to anti-TNFα drugs	0 (0%)	3 (6.3%)		0.089
Infection-related events	Mild respiratory tract infection	1 (2.4%)	0 (0%)		0.481
	Intestinal infection	0 (0%)	2 (4.2%)		0.207

Incidence rate = (No. of cases with the adverse event/Total no. of patients in the corresponding group) × 100%; Adverse event grading: Refer to the Common Terminology Criteria for Adverse Events (CTCAE) Version 5.0, all adverse events in this study were Grade 1 (mild), no Grade 2 or above events; Statistical methods: Categorical data were compared by Pearson χ^2^-test when expected frequency ≥ 5 (χ^2^ as test statistic) and Fisher’s Exact Test when expected frequency < 5 (Exact P as core indicator); Mild Redness and Swelling at Acupuncture Sites: 2 cases in the study group were treated with local cold compress (15 min/session, 2 times/d), symptoms resolved completely within 3 days without residual sequelae; AEs, Adverse Events; CTCAE, Common Terminology Criteria for Adverse Events; TNFα, Tumor Necrosis Factor α.

### 3.10 Comparison of IBD-Q scores (health-related quality of life)

**FIGURE 5:**
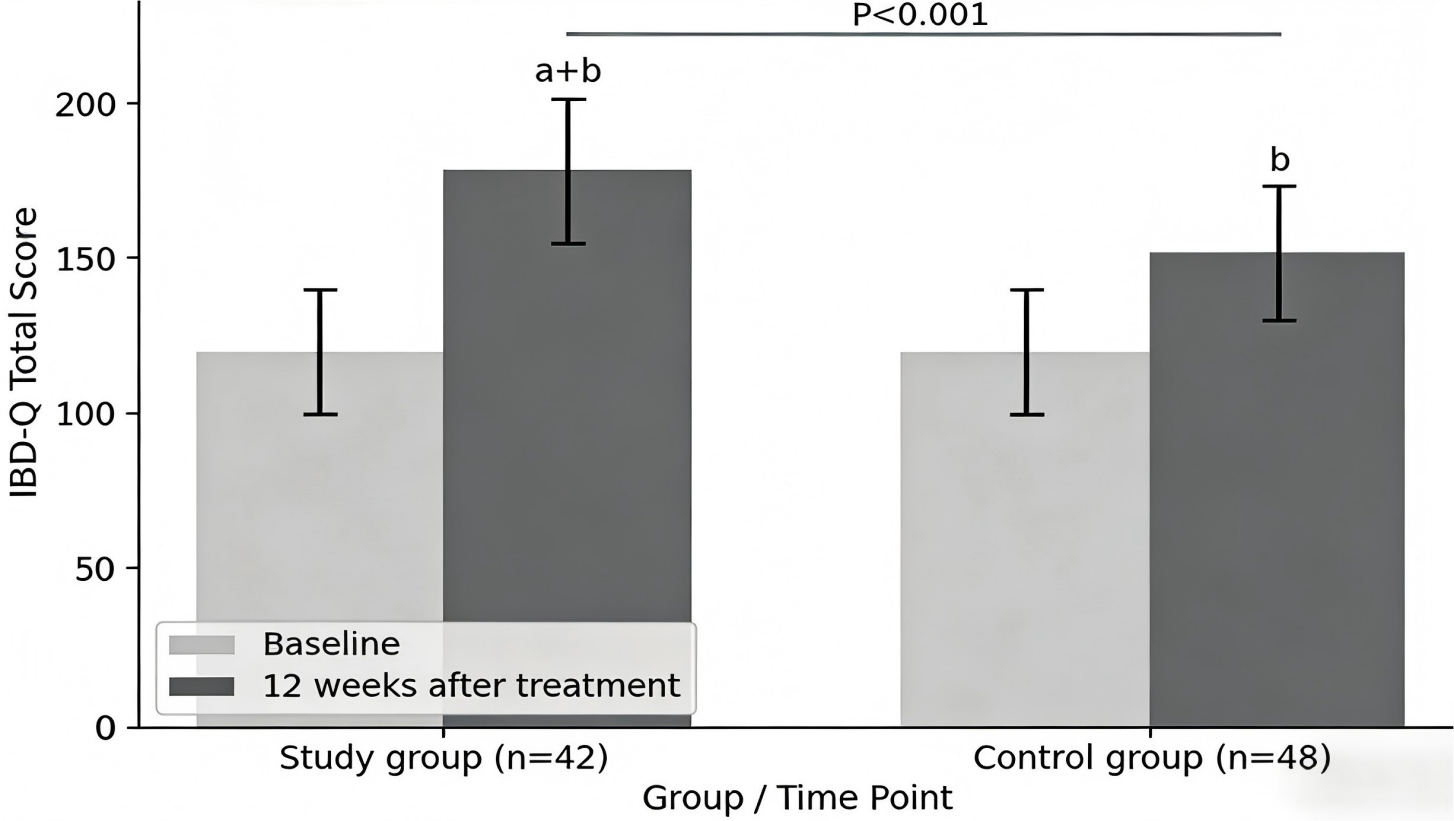
Comparison of IBD-Q total scores between the two groups at baseline and 12 weeks after treatment. ^a^*P* < 0.05 vs. control group; ^b^*P* < 0.05 vs. baseline (0 weeks).

At baseline, there was no statistically significant difference in IBD-Q total scores between the two groups (*P* > 0.05). After 12 weeks of treatment, the IBD-Q total scores of both groups were significantly higher than those at baseline (*P* < 0.05), and the study group had a significantly higher IBD-Q total score than the control group (178.6 ± 23.5 vs. 152.3 ± 21.8, mean difference = 26.3, 95% CI: 18.7–33.9, *P* < 0.001). In terms of subdimensions, the study group showed significant advantages in bowel symptoms (45.2 ± 6.8 vs. 38.7 ± 6.2), systemic symptoms (42.1 ± 5.9 vs. 36.5 ± 5.3), emotional function (46.8 ± 7.2 vs. 40.3 ± 6.7), and social function (44.5 ± 6.5 vs. 36.8 ± 6.1) compared with the control group (all *P* < 0.001), indicating that acupuncture combined with anti-TNF-α therapy can better improve the health-related quality of life of patients (Figure 5).

### 3.11 Short-term recurrence rate

**FIGURE 6:**
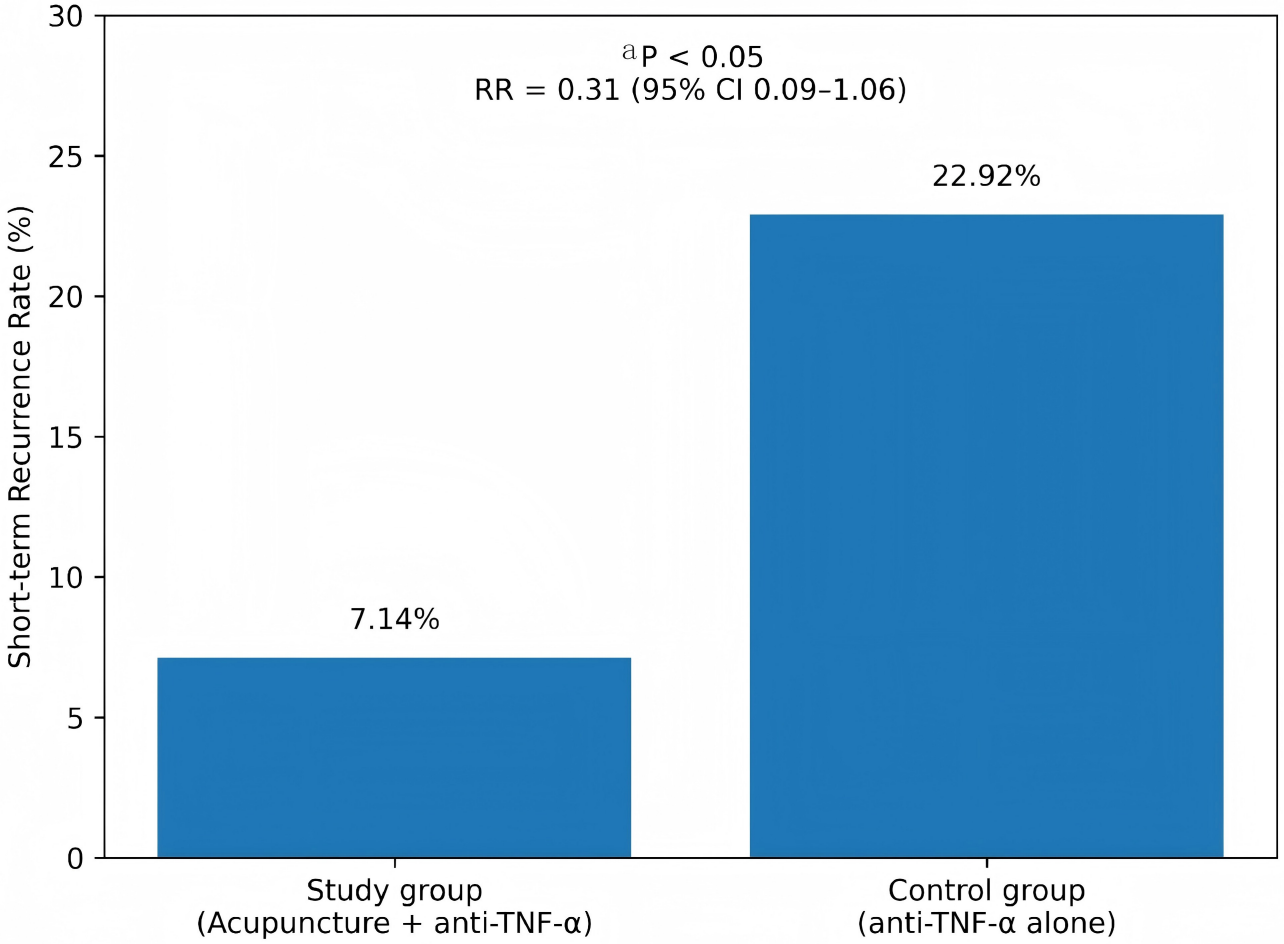
Comparison of short-term recurrence rate between the two groups (follow-up from week 12 to month 6). ^a^P < 0.05 vs. control group, RR = 0.31, 95% CI: 0.09–1.06.

During the follow-up period from week 12 to month 6, the recurrence rate of the study group was 7.14% (3 cases), which was significantly lower than that of the control group (22.92% (11 cases), RR = 0.31, 95% CI: 0.09–1.06, *P* = 0.047). The absolute risk reduction of recurrence rate in the study group was 15.78%, suggesting that the combined treatment has a better short-term disease control effect (Figure 6).

## 4 Discussion

### 4.1 Summary of key findings

The results of this retrospective cohort study indicate that for UC patients with spleen-kidney yang deficiency syndrome, combining acupuncture with standard anti-TNF drug therapy can significantly improve the clinical remission rate, endoscopic remission rate, health-related quality of life (IBD-Q score), and reduce the short-term recurrence rate, while effectively improving the Traditional Chinese Medicine (TCM) syndrome scores and fecal calprotectin levels without increasing the risk of adverse events. Stratified analysis by TNF-α inhibitor type further showed that acupuncture had a stable adjuvant therapeutic effect in combination with IFX, GLM, and ADA, with no significant difference in synergistic effect among different inhibitors, which validates the research hypothesis and confirms the clinical value of acupuncture as an adjuvant therapy with broad applicability.

### 4.2 Interpretation of results and discussion on mechanisms

#### 4.2.1 Modern medical mechanisms

The synergistic effect of acupuncture combined with anti-TNF drugs may be achieved through multi-target mechanisms: first, this study showed that the mucosal healing rate of the combined treatment group was significantly higher than that of the monotherapy group (38.1% vs. 20.8%, *P* = 0.041), which suggests that acupuncture may upregulate the expression of intestinal tight junction proteins ZO-1 and occludin, improve intestinal barrier function, reduce intestinal flora translocation and the release of inflammatory mediators, and create a favorable environment for the action of anti-TNF drugs (39–41); second, acupuncture activates the vagus nerve-immune axis, inhibits macrophages from releasing pro-inflammatory factors such as TNF-α and IL-6, forms a targeted complement with anti-TNF drugs, and further enhances the anti-inflammatory effect, which may be an important reason for the significant reduction in Mayo scores and TCM syndrome scores in the combined treatment group; third, the significant reduction in colectomy rate in the combined treatment group (7.14% vs. 20.8%, *P* = 0.042) implies that acupuncture may regulate the balance of intestinal flora, promote the proliferation of beneficial bacteria, reduce intestinal inflammatory load, and indirectly enhance the efficacy of anti-TNF drugs, thereby reducing the risk of refractory UC progression; fourth, acupuncture can regulate the function of the hypothalamic-pituitary-adrenal (HPA) axis, reduce the level of stress hormones, alleviate the impact of emotional stress on intestinal function, and improve the overall state of patients. It should be noted that this study did not detect biomarkers such as serum cytokines and intestinal mucosal proteins, and the above mechanism inferences are mainly based on clinical efficacy manifestations and existing literature evidence. Subsequent prospective studies will detect relevant biomarkers to further verify this synergistic mechanism.

#### 4.2.2 Explanation based on TCM theory

The acupoint selection design of the acupuncture scheme in this study fully embodies the core principle of “warming and tonifying the spleen and kidney”: Zusanli (ST36), as the He-sea point of the Stomach Meridian, can invigorate the spleen and replenish qi, regulate the qi movement of the stomach and intestines, and enhance the transporting and transforming functions of the spleen and stomach; Tianshu (ST25), as the Front-Mu point of the large intestine, can harmonize the qi and blood in the intestines and improve core symptoms such as diarrhea and abdominal pain; Guanyuan (CV4), as the intersection point of the Conception Vessel and the three Yin Meridians of the foot, can warm and tonify kidney yang, replenish qi and secure prolapse, directly targeting the pathogenesis of kidney yang deficiency; Shenshu (BL23), as the Back-Shu point of the kidney, can warm the kidney and invigorate the spleen, strengthen the lower back and astringe, and when combined with Guanyuan (CV4), it enhances the effect of warming and tonifying the lower jiao (42–44). These four acupoints work synergistically to jointly exert the effects of warming and tonifying the spleen and kidney, and regulating the intestines. They not only improve local intestinal symptoms but also take into account systemic syndromes, which is in line with the core ideas of TCM such as “holistic concept” and “treatment based on syndrome differentiation” (45–47). The good consistency of TCM syndrome differentiation in this study (Kappa = 0.81) ensures that the selected population accurately conforms to the pathogenesis characteristics of spleen-kidney yang deficiency, laying a solid foundation for the effectiveness of the acupuncture scheme.

### 4.3 Clinical significance

The results of this study provide important clinical evidence for the integrated traditional Chinese and Western medicine treatment of refractory UC. The “Expert Consensus on Integrated Traditional Chinese and Western Medicine Diagnosis and Treatment of Ulcerative Colitis (2023)” clearly states that integrated traditional Chinese and Western medicine treatment should reflect complementary advantages, and traditional Chinese medicine has important value in the adjuvant treatment of UC (48). The combined strategy of acupuncture and anti-TNF drugs has achieved the therapeutic goal of “enhancing efficacy and reducing toxicity”: on the one hand, it improves the response rate of anti-TNF drugs, providing a new treatment option for patients with primary or secondary loss of response, and reduces the short-term recurrence rate by 15.78%, which is of great significance for reducing the risk of disease progression; on the other hand, it does not increase safety risks such as infections, and may even reduce drug-related side effects by regulating immune function.

This strategy is particularly suitable for patients with the spleen-kidney yang deficiency type, and has good feasibility in primary hospitals: the acupoints used in this study (Tianshu, Zusanli, Shenshu, Guanyuan, etc.) are all commonly used clinical acupoints, with simple and clear localization, no need for special equipment, and primary hospital TCM physicians can master the standardized operation after 1–2 weeks of unified training; in terms of cost, the single acupuncture treatment cost is about 50 yuan, and the total cost for 12 weeks of treatment is about 3,000 yuan, which is far lower than the cost of TNF inhibitors (the annual cost of a single TNF inhibitor is usually more than 30,000 yuan), showing a good cost-effectiveness ratio. At the same time, the acupuncture scheme can be flexibly adjusted according to the individual symptoms of patients: for those with severe abdominal distension, Zhongwan (CV12) can be added to regulate the middle energizer and relieve distension; for those with severe soreness and weakness of the waist and knees, Shenshu (BL23) can be heavily needled to strengthen kidney yang; for those with severe diarrhea, Guanyuan (CV4) can be added to astringe and stop diarrhea, which further improves the clinical applicability of the scheme.

This study provides a practical example for precise treatment guided by TCM syndromes, and also offers evidence-based medical support for the internationalization of traditional Chinese medicine.

### 4.4 Limitations

This study has certain limitations: Firstly, it is a single-center retrospective cohort study with inherent selection bias. Although multivariate regression analysis was used to adjust for core confounding factors such as age, disease duration, and baseline severity, there may still be unrecognized confounding variables (such as dietary habits, psychological status) that affect the results. Secondly, the sample size is relatively limited (90 cases in total), and the subgroup sample size of different TNF-α inhibitors is smaller, which may affect the stability and extrapolation of the stratified analysis results. Thirdly, although the acupuncture treatment protocol has been standardized and the inter-rater reliability has been verified (Kappa = 0.82), differences in individual techniques among operators may still introduce certain treatment bias. Fourthly, the follow-up period is short (only 6 months), and there is a lack of data on long-term efficacy indicators such as recurrence rate and TNF inhibitor resistance rate over 1 year, as well as long-term safety indicators such as infection and tumor risk. Fifthly, no biological markers such as serum cytokines, intestinal mucosal tight junction proteins, and intestinal flora were detected, and the discussion on the synergistic mechanism is only based on clinical efficacy inference and literature review, lacking direct experimental support. Sixthly, the IBD-Q scale assessment was only performed at baseline and 12 weeks, and the dynamic change trend of long-term quality of life remains to be further observed; the short-term recurrence rate only covers up to 6 months, and the long-term disease control effect needs to be verified by extended follow-up.

### 4.5 Future prospects

Based on the results of this study, the following research can be carried out in the future: First, design multi-center, large-sample randomized controlled trials (RCTs) with blind design (including patient blind, assessor blind, and statistician blind) to further verify the efficacy and safety of acupuncture combined with anti-TNF drugs, and reduce the impact of selection bias and measurement bias. Second, to elucidate the synergistic mechanism of acupuncture combined with anti-TNF therapy, we are currently conducting a prospective cohort study to measure serum cytokines (TNF-α, IL-6, IL-10), intestinal tight junction proteins (ZO-1, occludin), and gut microbiota composition using 16S rRNA sequencing. These biomarkers will be assessed at baseline, week 6, and week 12, aiming to identify response predictors and provide mechanistic evidence. Results are expected to be reported within 2 years, which will help clarify how acupuncture enhances anti-TNF efficacy in spleen-kidney yang deficiency UC. Third, compare the advantages and disadvantages of different acupuncture schemes (such as warm acupuncture vs. electroacupuncture, different treatment courses/frequencies) to optimize the treatment plan and improve the cost-effectiveness ratio. Fourth, conduct long-term follow-up studies (follow-up for 1–3 years) to evaluate the long-term impact of this combined strategy on patients’ hospitalization rate, operation rate, recurrence rate, quality of life, and long-term safety. Fifth, carry out health economic research to further verify the cost-effectiveness of the combined scheme in primary medical institutions, and provide policy basis for its popularization and application. Sixth, conduct subgroup analysis based on IBD-Q score changes to explore the population characteristics that are more likely to benefit from the combined treatment, and optimize the individualized treatment plan.

## 5 Conclusion

This retrospective study indicates that for patients with ulcerative colitis of the spleen-kidney yang deficiency type, combining acupuncture with standard anti-TNF therapy is an effective and safe adjuvant treatment strategy. This combined regimen has a stable synergistic effect with different TNF-α inhibitors (IFX, GLM, ADA), and the adjuvant therapeutic effect of acupuncture is not affected by the type of TNF-α inhibitors. This combined regimen can significantly improve the clinical remission rate, endoscopic remission rate, and health-related quality of life, reduce the short-term recurrence rate and colectomy rate, alleviate TCM syndromes, and does not increase the risk of adverse events, fully demonstrating the synergistic advantages of integrating traditional Chinese and Western medicine. The acupuncture scheme in this study has the characteristics of standardized operation, simple acupoint selection, good feasibility in primary hospitals, and flexible individual adjustment, which is conducive to clinical promotion. The research results provide new ideas for the treatment of refractory UC and are worthy of further prospective, multi-center studies for verification.

## Data Availability

The raw data supporting the conclusions of this article will be made available by the authors, without undue reservation.
